# The Strange Case of the Rev. Mr. Hanna

**Published:** 1905-02

**Authors:** 


### THE STRANGE CASE OF THE REV. MR. HANNA.

Every mature reader of Robert Louis Stevenson's uncanny portrayal of the dual personality of Dr. Jekyll and Mr. Hyde must have reflected that there is something strangely true to life in this tale; that in every individual there seem to be two natures, oftentimes antagonistic the one to the other. In the now almost obsolete works of Carpenter, these phenomena were attributed to the lateral halves of the brain sometimes working independently of one another, the more powerful half dominating the organism, while the other remained impotent.

We have now a scientific consideration of a very striking case by Dr. Boris Sidis in the book entitled, *Multiple Personality: An Experimental Investigation into the Nature of Human Individuality.* In this work, Dr. S. P. Goodhart is a collaborator.*

The Rev. C. Hanna, while returning from a drive on the evening of April 15, 1897, "stepped aside" from the accustomed order of things by plunging headlong from his carriage, and thus "became, strangely enough, a man endued with preternatural intelligence, but from whose mind all knowledge of human and terrestrial relations had subsided."

On awakening to the new personality, a terrific encounter took place with his physicians. He could not recognize them as his fellow-men. Now fully recovered, he remembers distinctly the events in this crisis of his life. In his autobiographical sketch he declares: "The first return to consciousness may be understood only by comparing it to the birth of a person possessed immediately of matured mental and physical functions." During the first rudiments of consciousness there was absolute lack of knowledge that an outside world was in existence. The eyes were closed; there were no sounds to be heard; the power of motion was not yet known. We quote him:

"Simple memory would represent it as a period of many years, so great was the mental activity and so wonderful were even the meagre facts of consciousness. But the fact that absolutely no motion was made, even of the eyelids, and that no sound was heard, although the room was full of watchers, apparently indicates that the state was of but an instant's duration. In fact, later experience which left on me the impression that would now be made by the life-happenings of many years, really continued only for a few moments, according to the positive testimony of others who observed them. Thus, if memory, with its present habits of comparison, was to be trusted for a verdict, my life for the first few days would be declared to cover a few centuries, and this impression has been corrected only by force of the most vigorous reasoning on my part and the exercise of the perfect power of the will in forming beliefs."

*Publ. D. Appleton & Co'

We find here, in passing, an odd confirmation of the Kantian dictum that time and space are but *a priori conceptions* of reason, in no wise related to experience; also of the fact that in dreams, happenings that would consume long periods of time are conceived in a single instant.

In his former state Mr. Hanna was a fine scholar, an architect, a journalist, an excellent musician, and a thorough musician. And we hasten to add that no Mr. Hyde-like propensities of an evil sort occurred during his peculiar experience. He was twenty-three at the time of the accident and was then attending to his ministerial duties. He was of good family, sociable, well liked by his congregation, and was an altogether sensible and normal man.

In his new state his eyes were opened to a world of moving shadows. He found himself capable of movement; the shadows he thought a part of himself. He put forth his arm, which came in contact with one of them, and to his alarm, it resisted. The shadows multiplied and the force against him redoubled. He had, in fact, attacked his physicians with herculean strength, and in the struggle he worsted them. He was, however, finally bound, and put to bed. No one yet understood his predicament—from his viewpoint—and they thought him insane from the blow on the head received by his fall. He recognized neither objects, words, or persons, nor dimensions—length, breadth and depth; nor the color of objects. External things formed a great picture against his eyes like a painting, with no sense of spatial relations. There was no conception of the flow of time. External movement alone fastened his voluntary attention, and seemed to fascinate his gaze. He became intensely hungry, but could not interpret his desire; he had to be fed by fluids placed far back into the pharynx.

He could not understand human speech. But after the struggle with the doctors, his intellect and perception, sharpened by the precariousness of his position, grasped the notion that there was a difference in persons, and that by lip movements and sound productions, they elicited from each other responses which were frequently followed by definite actions. Without even slightly knowing their meaning, he imitated their suggestions and observed that thereby he attracted attention.

They had to teach him to talk like a child. He made great progress, learning first the names of concrete objects, and very soon the expression of abstract ideas. In six weeks the secondary personality was so trained and educated, that he could explain lucidly what had taken place since the accident. He naïvely described his discovery of himself as a human being. He had been kept in bed and those about him were dressed. "I said people;" that meant them all. Mr. S. told me that I was people, and he told me I was a man—same as he was; and I did not believe that. I began to ask him why I could not have clothes on like other people. That was after I had learned to talk a great deal."

It was difficult for him to differentiate the sexes; they were "two strange kinds of human beings." He was engaged to a lady, whom in his secondary state he did not know. He was questioned whether any subconscious feeling of recognition and love could be revealed:

Q.—Do you enjoy rather to look at a nice-looking woman than at a nice-looking man? A.—I do not know; it does not make so much difference. If it is a picture, they must be very nice-looking to enjoy, but if it is a person, I like to hear the voice. The voices of women are softer and more pleasant to hear.

Q.—Would you have any desire to kiss or embrace a woman whom you liked very much? A.—Yes, my mother or sister.

With life outdoors his mental horizon expanded amazingly, and the new personality became speedily accoutred with all the aids of prolonging and preserving its identity in the world. But once his mind let go its hold on some unimportant matter, and for the first time in his new existence he learned what it meant to forget. This profoundly impressed him and made him comprehend the references by friends to his obliviousness of his former life. Then the well-directed efforts of his physicians resulted in disconnected pictures, scenes in his previous existence being aroused in his dreams while sleeping. From this time their efforts centered on restoring the primary personality. Among many experiments that were made was this: During a restless early morning sleep, questions were put to him in his half-awakened state, and his answers showed that he was dreaming over old scenes. He was describing a climb up

Mount Jewett, when suddenly he was asked if he knew his fiancée. He laughed, regarding the question as irrelevant, and said, "I don't know her yet; I know her later. From her to Mount Jewett is a year." This was a correct statement. The personality was in a peculiar psychic state, in which it was able to foresee its future life. It was as if there were prescience of what was to take place in the distant future.

Mr. Hanna was one evening taken to New York where the stimulation of new surroundings had the effect that early the following morning he recovered his submerged personality. He described his ride from Meriden, Conn., on the night of the accident, and complained of soreness and exhaustion, which he ascribed to his fall of "last Thursday," the accident having, in fact, occurred some seven weeks before. He was told of his secondary existence, of which he was ignorant, and within an hour, elapsed again into that state.

Then came the strangest feature in this case, which strongly resembled that in Stevenson's story. Dr. Jekyll, we recall, was restored by a powder, which Hyde took, until finally the more powerful personality of Hyde prevailed. A drug (cannabis indica) was also used in the case of Mr. Hanna, but with this difference —that partly by the unremitting efforts of his physicians, and partly by his own will, his original personality gradually gained reascendency over the second nature and won the psychic struggle.

In his dual life Mr. Hanna alternated until on June 14, while visiting a psychological laboratory, he for the first time caught a glimpse of both personalities simultaneously, and yielded to the inducements of his second nature. His responses to persistent questioning during the transformation, "indicated now the presence of the primary, now that of the secondary state, and now of the simultaneous presence of both." There was an intense mental struggle, which was fought out that afternoon, where he lay, on a couch in his physician's office, "in a state of agonized abstraction. It was a bitter and a critical period of his life." Two alien individuals had arisen in his consciousness to dispute the possession of his mind, each claiming to be his veritable self. To renounce either was to blot out his own existence. The result was, a reconcilement of both and their fusion. States Dr. Sidis:

"These two formed individualities, seemingly mortal foes, confronted each other for a long period of time, and in their very struggle recognized their intimate relationship, if not their relationships of identity. It seemed as if each said to the other: 'Thou art my mortal foe, and yet thou art bone of my bones and flesh of my flesh!' for each personality to crush, to suppress the other, was now out of the question: the difficulty, the problem, for them, was how to form a unity; how to become synthetized into one conscious personality. The task was a difficult one, and could be achieved at a loss of much mental energy. Hence, the sluggishness of psychomotor activity, the slowness of movement, of speech, of reactions to external stimuli; hence, the retardation of the whole stream of consciousness," etc. Mr. Hanna has fully recovered, the detached portions have become dovetailed, the two sharply-defined personalities have been fused into one normal personality.—*New York Medical Times.*

